# Skull metastasis is a poor prognostic factor for prostate cancer patients with bone metastasis: a retrospective study based on a Chinese population

**DOI:** 10.1186/s12894-023-01179-9

**Published:** 2023-01-31

**Authors:** Tianyu Xiong, Mingxin Jiang, Xiaobo Ye, Guangyi Zhu, Fang Cao, Yun Cui, Minfu Yang, Yinong Niu

**Affiliations:** 1 Department of Urology, Beijing Chaoyang Hospital, Capital Medical University, Beijing, China; 2 Department of Urology, Beijing Shijitan Hospital, Capital Medical University, 10 Tieyiyuan Road, Haidian District, Beijing, China; 3 Department of Nuclear Medicine, Beijing Chaoyang Hospital, Capital Medical University, Beijing, China

**Keywords:** Skull metastasis, Prostate cancer, Bone metastasis, Prognosis, Castration-resistant prostate cancer

## Abstract

**Background:**

Skull is a relatively rare metastasis site for prostate cancer (PCa). There is no evidence regarding the prognostic indication of skull metastasis (SM) in PCa patients. In this study, we analyzed the prognostic value of SM for metastatic PCa patients receiving androgen deprivation therapy (ADT).

**Methods:**

107 consecutive patients were included from September 2008 to August 2021. All patients were administered with standard ADT. Abiraterone plus glucocorticoid and/or docetaxel chemotherapy were given after failure to castration-resistant prostate cancer. Clinical parameters and follow-up prognostic data were retrospectively analyzed. The association of clinical and pathological parameters with SM were analyzed. The progression-free survival (PFS) and overall survival (OS) were assessed using Kaplan–Meier analysis and Cox regression analyses.

**Results:**

Patients with SM (n = 26) had significantly higher biopsy Gleason scores, higher clinical T stage, higher prostate-specific antigen level at diagnosis, and were more likely to have high-burden metastasis and lymph node metastasis, compared with those without SM (n = 81). They also showed significantly lower level of hemoglobin, albumin and serum calcium, along with higher level of alkaline phosphatase. SM was significantly associated with shorter medium PFS (9.4 vs*.* 18.3 months, *p* < 0.001) and OS (22.2 vs*.* 58.2 months, *p* < 0.001). Cox analysis demonstrated that SM was an independent risk factor for shorter PFS (hazard ratio 2.327 [1.429–3.789], *p* = 0.001) and shorter OS (hazard ratio 2.810 [1.615–4.899], *p* < 0.001).

**Conclusion:**

In this study, we found that SM was significantly correlated with more aggressive disease and indicated poor prognosis in PCa patients with bone metastasis. Our study may provide useful reference for the risk stratification of PCa patients.

**Supplementary Information:**

The online version contains supplementary material available at 10.1186/s12894-023-01179-9.

## Background

Prostate cancer (PCa) is the second most common malignant disease in males and accounted for over 360,000 death globally in 2018 [1]. For PCa patients, bone metastasis is correlated with poor prognosis and increasing economical burden [2–5]. The extent of bone metastases has been confirmed to be associated with poor prognosis for PCa patients [6–9]. Quantitative or semi-quantitative parameters, such as the extent of disease (EOD) score and Bone Scan Index, have been adopted to reflect disease aggressiveness and predict the prognosis for metastatic PCa patients. However, studies on the different prognostic value among various bone metastasis sites are still limited.

Skull is a relatively rare metastasis site for most kinds of solid cancers [10]. Noticeably, literature reviews showed a relatively higher metastatic rate to skull in PCa cancer patients [11–15]. As a distant metastasis site from the primary tumor, skull metastasis (SM) may be a sign of more aggressive disease and shorter survival rate after treatment [16–18]. However, the prognostic implication of SM in PCa patients is still not clear.

In this study, we analyzed whether SM was correlated with more advanced disease based on a group of Chinese PCa patients. We also evaluated whether SM was an independent risk factor for castration-resistant prostate cancer (CRPC) and death after systemic treatment.

## Methods

### Patient selection and data collection

We retrospectively reviewed data of PCa patients with bone metastasis in Beijing Chaoyang Hospital from September 2008 to August 2021. All patients were diagnosed of PCa by ultrasound guided transrectal prostate biopsy. Bone metastasis was identified by bone scan, magnetic resonance imaging (MRI) or ^18^F-fluorodeoxyglucose positron emission tomography/computed tomography (PET/CT). The following exclusion criteria were used: (1) incomplete data, (2) any visceral metastasis indicated at initial diagnosis, (3) received any type of anticancer therapy before the starting point of observation. Our study was conducted in accordance with the Declaration of Helsinki (as revised in 2013) and was approved by the Institutional Review Board of Beijing Chaoyang Hospital, Capital Medical University (NO.: 2022-Ke-55), which waived the requirement of informed consent for this retrospective analysis.

Clinical tumor stage, presence of lymph node metastasis, biopsy Gleason score, serum prostate-specific antigen (PSA) level, age, body mass index (BMI) and other clinical parameters at initial diagnosis were retrieved from the electronic medical records. Images of MRI, bone scan or PET/CT prior to any treatment were reviewed to evaluate the presence of SM and number of bone metastasis lesions. The EOD score was categorized according to the definition reported by Soloway et al. [6]. EOD score ≥ 2 cases (6 or more bone metastasis lesions or “Super bone scan”) were defined as high-burden metastasis. The endpoints of this study were CRPC and death. CRPC was defined as the status in which PCa progressed clinically, radiographically or biochemically despite castration levels of serum testosterone (< 50 ng/dL). The progression-free survival (PFS) time was calculated from the date of diagnosis of metastatic PCa to the date of CRPC or death (if CRPC status was not confirmed). The overall survival (OS) was defined as time from the start of observation to death.

### Treatments

All patients were initially treated with standard androgen deprivation therapy, which was administered with androgen receptor blocker (bicalutamide) and GnRH agonist (leuproline or goserelin), or surgical castration with an antiandrogen. After failure to CRPC, the patients were given abiraterone plus glucocorticoid (prednison or dexamethasone), docetaxel chemotherapy, or both. For terminally ill patients, pain relief and palliative radiotherapy were used as appropriate. The use of bone modifying agents including bisphosphonate and denosumab was not regulated.

### Statistical analyses

In this study, continuous variables were expressed as median with interquartile ranges and compared using the Mann–Whitney *U* test. Categorical variables were compared using the chi-square test or Fisher’s exact test as appropriate. Kaplan–Meier method was used to estimate the medium of PFS and OS. The log-rank test was used for analysis of the survival differences between patients with or without SM. Cox regression analysis were used to calculate the respective hazard ratio and 95% confidence intervals for the SM and other clinical and pathological parameters. Variables found to be significant in univariate analysis (*p* < 0.1) were entered into the multivariate analysis. SPSS version 26.0 (IBM Corp., Armonk, NY, USA) and R version 4.1.2 (http://www.r-project.org/) was utilized for statistical analysis. All statistical tests were two-tailed, and a *p* value < 0.05 was considered significant for all parameters.

## Results

### Baseline characteristics

A total of 107 consecutive patients were included in this study. 26 (24.3%) patients were identified as SM and categorized into the SM group and the other 81 (76.4%) patients were categorized into the non-SM group. The baseline clinical and pathological characteristics of all patients were presented in Table 1. Patients with SM had significantly higher clinical T stage (cT3 stage 46.2% vs*.* 28.4%, cT4 stage 42.3% vs*.* 32.1%, *p* = 0.027), more lymph node metastasis (65.4% vs. 42.0%, *p* = 0.038), more high-burden metastasis (EOD score ≥ 2) (96.2% vs. 29.6%, *p* < 0.001) and higher PSA level at diagnosis (758.55 [427.25–950.99] ng/mL vs. 131.75 [35.35–344.31] ng/mL, *p* < 0.001). Additionally, patients in the SM group showed significantly lower level of hemoglobin (119 [107–133] g/L vs. 130 [118–142] g/L, *p* = 0.029), albumin (37.6 [33.8–41.2] g/L vs. 41.1 [38.3–43.4] g/L, *p* = 0.006) and serum calcium (2.20 [2.06–2.30] mmol/L vs. 2.26 [2.17–2.36] mmol/L, *p* = 0.035), accompanied by higher level of alkaline phosphatase (315 [161–567] u/L vs. 94 [78–162], *p* < 0.001).

**Table 1:** Baseline characteristics of all patients

Factors	Total (n = 107)	Non-skull metastasis group (n = 81)	Skull metastasis group (n = 26)	*p* value
Age at diagnosis (year), median (IRQ)	73 (67–79)	74 (67–80)	71 (67–75)	0.108
BMI at diagnosis (kg/m^2^), median (IRQ)	23.77 (21.81–25.61)	23.89 (22.15–26.03)	22.41 (20.83–24.98)	0.079
Clinical T stage, n (%)				0.027
T2	35 (32.7)	32 (39.5)	3 (11.5)	
T3	35 (32.7)	23 (28.4)	12 (46.2)	
T4	37 (34.6)	26 (32.1)	11 (42.3)	
Clinical N stage, n (%)				0.038
N0	56 (52.3)	47 (58.0)	9 (34.6)	
N1	51 (47.7)	34 (42.0)	17 (65.4)	
High-burden metastasis, n (%)	49 (45.8)	24 (29.6)	25 (96.2)	< 0.001
PSA level at diagnosis (ng/ml), median (IRQ)	212.61 (43.44–745.66)	131.75 (35.35–344.31)	758.55 (427.25–950.99)	< 0.001
Biopsy Gleason score, n (%)				0.899
No more than 8	34 (31.8)	26 (32.1)	8 (30.8)	
9 or 10	73 (68.2)	55 (67.9)	18 (69.2)	
Hemoglobin (g/L), median (IRQ)	129 (116–140)	130 (118–142)	119 (107–133)	0.029
Albumin (g/L), median (IRQ)	40.3 (36.3–43.3)	41.1 (38.3–43.4)	37.6 (33.8–41.2)	0.006
Serum calcium (mmol/L), median (IRQ)	2.24 (2.15–2.36)	2.26 (2.17–2.36)	2.20 (2.06–2.30)	0.035
Alkaline phosphatase (u/L), median (IRQ)	105 (84–202)	94 (78–162)	315 (161–567)	< 0.001

IQR, interquartile range; BMI, body mass index; PSA, prostate-specific antigen

### Survival outcomes

**Fig. 1:**
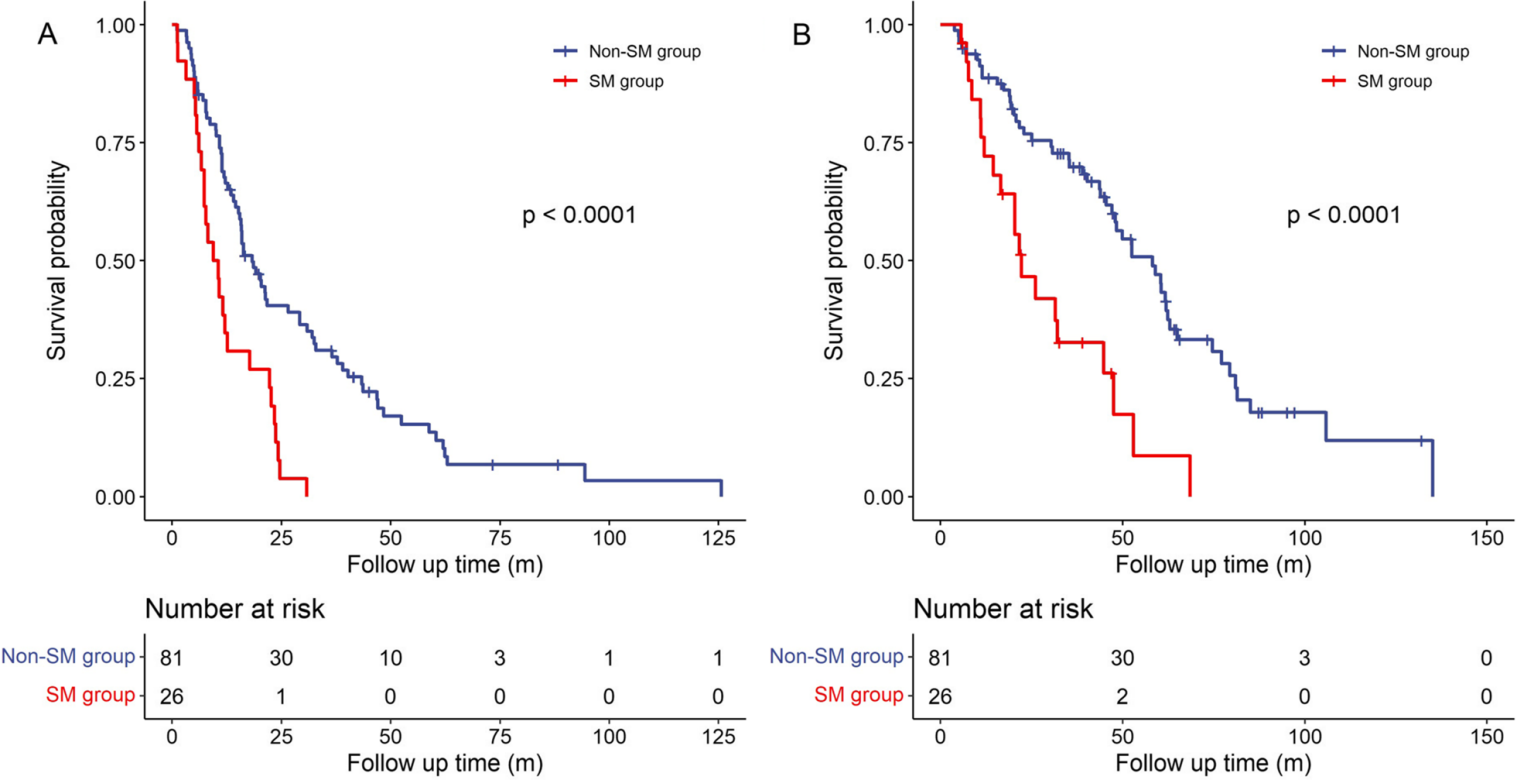
Kaplan–Meier analyses of PFS and OS time between the SM group and non-SM group: **A** Analysis of PFS time, **B** analysis of OS time. PFS, progression free survival; OS, overall survival; SM, skull metastasis

The medium follow-up time was 36.5 months (interquartile range 18.9–58.9 months). Kaplan–Meier curve analysis showed SM was significantly associated with shorter medium PFS (9.4 months vs. 18.3 months, *p* < 0.001) and OS time (22.2 months vs. 58.2 months, *p* < 0.001), as shown in Fig. 1. Among patients with high-burden metastasis, those with SM also showed shorter medium PFS (10.6 months vs. 15.9 months, *p* = 0.005) and OS time (22.2 months vs. 47.9 months, *p* = 0.041) (Additional file 1: Figure S1). Univariable Cox analysis showed that SM, high-burden metastasis, clinical stage (T4), lymph node metastasis and anemia were significantly associated with shorter PFS (all *p* values < 0.05). Based on multivariable analysis, SM (hazard ratio 2.327 [1.429–3.789], *p* = 0.001) and clinical stage (T4) (hazard ratio 1.627 [1.055–2.509], *p* = 0.028) were significantly associated with shorter PFS. For OS time, univiarate Cox regression analysis showed that SM, high-burden metastasis, clinical stage (T4) and anemia showed significant association (all *p* values < 0.05). In multivariable analysis, SM (hazard ratio 2.810 [1.615–4.899], *p* < 0.001) and clinical stage (T4) (hazard ratio 1.639 [1.003–2.679], *p* = 0.049) were independent risk factors for OS (Table 2).

**Table 2:** Univariate and multivariate Cox regression analyses of PFS and OS time

Variables	PFS				OS			
	Univariate analysis		Multivariate analysis		Univariate analysis		Multivariate analysis	
	HR (95% CI)	*p* value	HR (95% CI)	*p* value	HR (95% CI)	*p* value	HR (95% CI)	*p* value
Skull metastasis	2.545 (1.572–4.121)	< 0.001	2.327 (1.429–3.789)	0.001	2.892 (1.666–5.023)	< 0.001	2.810 (1.615–4.899)	< 0.001
Age > 70 years	1.310 (0.747–2.298)	0.398			1.242 (0.758–2.036)	0.390		
BMI ≥ 25 kg/m^2^	1.056 (0.676–1.650)	0.811			1.034 (0.626–1.707)	0.896		
High-burden metastasis	1.516 (1.011–2.274)	0.044	1.040 (0.621–1.741)	0.883	1.736 (1.084–2.779)	0.022	1.232 (0.684–2.219)	0.488
PSA > 100 ng/mL	0.872 (0.576–1.318)	0.515			0.817 (0.507–1.317)	0.407		
Clinical T4 stage	1.813 (1.184–2.776)	0.006	1.627 (1.055–2.509)	0.028	1.710 (1.048–2.790)	0.032	1.639 (1.003–2.679)	0.049
Clinical N1 stage	1.560 (1.039–2.341)	0.032	1.174 (0.746–1.848)	0.488	1.238 (0.773–1.983)	0.374		
Biopsy Gleason score > 8	1.315 (0.848–2.038)	0.221			0.847 (0.513–1.399)	0.516		
Hemoglobin level at diagnosis < 120 g/L	1.538 (1.024–2.448)	0.044	1.279 (0.803–2.038)	0.300	1.639 (0.996–2.696)	0.052	1.283 (0.749–2.200)	0.364
Albumin < 40 g/L	0.848 (0.566–1.271)	0.425			0.761 (0.468–1.236)	0.270		
Serum calcium < 2.2 mmol/L	0.757 (0.504–1.137)	0.180			0.874 (0.544–1.404)	0.578		
Alkaline phosphatase > 125 u/L	1.348 (0.899–2.020)	0.148			1.117 (0.696–1.793)	0.646		

HR, hazard ratio; CI, confidence interval; PFS, progression free survival; OS, overall survival; BMI, body mass index; PSA, prostate-specific antigen

## Discussion

In the present study, we found that SM was a poor prognosis factor for PCa patients with bone metastasis based on the data of a Chinese population. SM was significantly associated with higher biopsy Gleason scores, higher clinical T stage, higher PSA level at diagnosis, and were more likely to have high-burden metastasis and lymph node metastasis. Patients with SM had significantly short PFS and OS, reflecting a poor response to ADT. In addition, patients in the SM group showed significantly lower level of hemoglobin, albumin and serum calcium, and higher level of alkaline phosphatase at initial diagnosis, which suggested the nutrition status of patients with SM might be compromised. These results indicated that patients with SM had more aggressive disease and poor prognosis.

Bone is the most common site of PCa metastases and involved in approximately 90% of metastatic PCa patients [19]. This remarkable metastasis rate of PCa may be partly explained by a high affinity to bones, which is also called the "dependence of the seed on a fertile soil" hypothesis [20]. The development of bone metastasis is a comprehensive multi-step process, including the colonization of circulating cancer cells and reconstruction of bone structure and function [21, 22]. Unlike other types of malignant diseases, bone metastasis originated from PCa uniquely induces bone formation, which can be detected by bone scan or biopsy [23].

Spine, pelvis and ribs are the most frequently observed sites of bone metastasis in PCa patients [11]. On the other hand, a gradual decrease in spine involvement from the lumbar to the cervical part was observed in a study based on autopsy results. Anatomical factors, such as the venous Batson’s plexus along the spine, may explain this high rate of probability axial bone involvement [24, 25]. The association between bone metastases on the appendicular skeleton (ribs and limbs) and shorter overall survival in PCa patients has been reported by Rigaud et al. [26]. As skull is one of the most distant site of possible metastasis, we believe SM is also a reflection of high aggressiveness in PCa patients. Our hypothesis was supported by the results of our study, which SM was an independent risk factor for shorter PFS and OS. To our knowledge, our study was the first to report SM as a poor prognostic factor for PCa patients.

There were some limitations in our study. First, it was a retrospective and single-institution study, resulting in potential selection bias. Second, due to a relatively small sample size, we were unable to differentiate the prognostic effect of SM among patients with low-burden metastasis, as we did for those with high-burden metastasis. Third, the diagnosis of SM was limited on accuracy. Fourth, we could not retrieve the data of cranial nerve palsies which was reported to indicate a much shorter survival [27].

In conclusion, we found SM was correlated with poor prognosis for PCa patients with bone metastasis. Our study may provide useful reference for the risk stratification of PCa patients.

## Supplementary Information

**Additional file 1. Figure S1.** Kaplan-Meier analyses of PFS and OS time among patients with high-burden metastasis (6 or more bone metastasis lesions or “Super bone scan”): (A) Analysis of PFS time, (B) Analysis of OS time. PFS: progression free survival; OS: overall survival; SM: skull metastasis.

## Data Availability

The clinical and follow-up data used to support the findings of this study are restricted by the Institutional Review Board of Beijing Chaoyang Hospital, Capital Medical University, in order to protect the patient privacy. Data are available from Yinong Niu (E-mail: niuyinong@mail.ccmu.edu.cn) for researchers who meet the criteria for access to confidential data.

## Abbreviations

ADT: Androgen deprivation therapy
BMI: Body mass index
CRPC: Castration-resistant prostate cancer
EOD: Extent of disease
MRI: Magnetic resonance imaging
OS: Overall survival
PCa: Prostate cancer
PET/CT: Positron emission tomography/computed tomography
PFS: Progression free survival
PSA: Prostate-specific antigen
SM: Skull metastasis
