# Supplementary material for: Skull metastasis is a poor prognostic factor for prostate cancer patients with bone metastasis: a retrospective study based on a Chinese population
Source: BMC Urol. 2023 Jan 31;23:13. doi: 10.1186/s12894-023-01179-9 (PMC9887768; doi:10.1186/s12894-023-01179-9)
Supplement: Supplementary file 1 — Additional file 1. Figure S1. Kaplan-Meier analyses of PFS and OS time among patients with high-burden metastasis (6 or more bone metastasis lesions or “Super bone scan”): (A) Analysis of PFS time, (B) Analysis of OS time. PFS: progression free survival; OS: overall survival; SM: skull metastasis. [file 12894_2023_1179_MOESM1_ESM.docx]

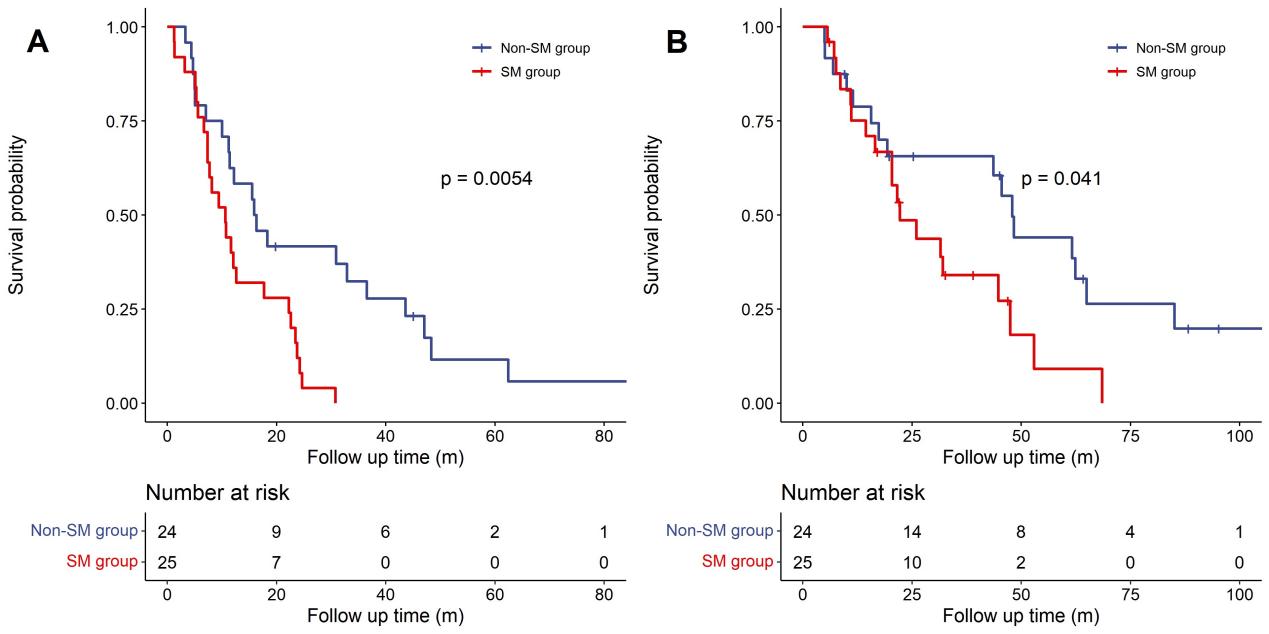


Supplementary Figure 1 Kaplan-Meier analyses of PFS and OS time among patients with high-burden metastasis (6 or more bone metastasis lesions or “Super bone scan”): (A) Analysis of PFS time, (B) Analysis of OS time. PFS: progression free survival; OS: overall survival; SM: skull metastasis.
